# Supplementary material for: Decline of German and rise of North-American hegemony in science: Insights from Nobel Prize nominations (Physics/Chemistry, 1901–1969)
Source: PLoS One. 2025 May 8;20(5):e0323103. doi: 10.1371/journal.pone.0323103 (PMC12061115; doi:10.1371/journal.pone.0323103)
Supplement: S1 Table — Standard errors in brackets: * p < 0.05, ** p < 0.01, *** p < 0.001. (DOCX) [file pone.0323103.s001.docx]

**Table S1: Logistic regression model for making a successful nomination**

|  | Model 1 | Model 2 | Model 3 |
| --- | --- | --- | --- |
| **Nominees‘ variables** |  |  |  |
| Country |  |  |  |
| Reference category: all other countries |  |  |  |
| Germany | 0.50***  (0.10) | 0.70***  (0.11) | 0.69***  (0.12) |
| USA | 0.42***  (0.08) | 0.64***  (0.10) | 0.63***  (0.10) |
| UK | - | 0.76***  (0.12) | 0.74*** (0.13) |
| FR | - | - | -0.05  (0.17) |
| **Nominators‘ variables** |  |  |  |
| Country |  |  |  |
| Reference category: all other countries |  |  |  |
| Germany | -0,39***  (0.06) | -0,41***  (0.11) | -0.42***  (0.11) |
| USA | -0.27***  (0.10) | -0.33***  (0.10) | -0.34***  (0.10) |
| UK | - | -0.38**  (0.15) | -0.38**  (0.15) |
| France | - | - | -0.02  (0.14) |
| N | 8110 | 8110 | 8110 |
| McFadden Pseudo R² | 0.01 | 0.01 | 0.01 |
| Nagelkerke Pseudo R² | 0.01 | 0.02 | 0.02 |
| BIC | 5928 | 5909 | 5927 |
| AIC | 5893 | 5860 | 5864 |

Standard errors in brackets: ^*^ *p* < 0.05, ^**^ *p* < 0.01, ^***^ *p* < 0.001
