# Supplementary material for: Decline of German and rise of North-American hegemony in science: Insights from Nobel Prize nominations (Physics/Chemistry, 1901–1969)
Source: PLoS One. 2025 May 8;20(5):e0323103. doi: 10.1371/journal.pone.0323103 (PMC12061115; doi:10.1371/journal.pone.0323103)
Supplement: S2 Table — Standard errors in brackets: * p < 0.05, ** p < 0.01, *** p < 0.001. (DOCX) [file pone.0323103.s002.docx]

**Table S2: Logistic regression models (time periods) for making a successful nomination**

|  | Model 1  1911-1933 | Model 2  1911-1933 | Model 3  1911-1933 | Model 1  1934-1969 | Model 2  1934-1969 | Model 3  1934-1969 |
| --- | --- | --- | --- | --- | --- | --- |
| **Nominees‘ variables** |  |  |  |  |  |  |
| Country |  |  |  |  |  |  |
| Reference category: all other countries |  |  |  |  |  |  |
| Germany | 0.77***  (0.15) | 0.92***  (0.16) | 0.83***  (0.19) | -0.17  (0.17) | 0.08  (0.18) | 0.05  (0.18) |
| USA | -0.11  (0.26) | 0.01  (0.28) | -0.07  (0.29) | 0.51***  (0.10) | 0.78***  (0.11) | 0.75***  (0.12) |
| UK | - | 0.63*  (0.29) | 0.56  (0.30) | - | 0.83***  (0.14) | 0.80***  (0.14) |
| France | - | - | -0.13  (0.27) | - | - | -0.17  (0.24) |
|  |  |  |  |  |  |  |
| **Nominators‘ variables** |  |  |  |  |  |  |
| Country |  |  |  |  |  |  |
| Reference category: all other countries |  |  |  |  |  |  |
| Germany | -0.43**  (0.15) | -0.41**  (0.12) | -0.44**  (0.16) | -0.41**  (0.15) | -0.45**  (0.15) | -0.45**  (0.15) |
| USA | -0.34  (0.28) | -0.35  (0.29) | -0.38  (0.28) | -0.23*  (0.10) | -0.30**  (0.10) | -0.30**  (0.11) |
| UK | - | 0.45  (0.32) | 0.41  (0.32) | - | -0.54**  (0.17) | -0.54**  (0.17) |
| France | - | - | -0.16  (0.25) | - | - | 0.05  (0.16) |
|  |  |  |  |  |  |  |
| N | 1932 | 1932 | 1932 | 6178 | 6178 | 6178 |
| McFadden Pseudo R² | 0.02 | 0.03 | 0.03 | 0.01 | 0.02 | 0.02 |
| Nagelkerke Pseudo R² | 0.03 | 0.04 | 0.04 | 0.02 | 0.03 | 0.03 |
| AIC | 1609 | 1601 | 1604 | 4225 | 4191 | 4195 |
| BIC | 1637 | 1640 | 1654 | 4258 | 4239 | 4256 |

Standard errors in brackets: ^*^ *p* < 0.05, ^**^ *p* < 0.01, ^***^ *p* < 0.001
