# Supplementary material for: Decline of German and rise of North-American hegemony in science: Insights from Nobel Prize nominations (Physics/Chemistry, 1901–1969)
Source: PLoS One. 2025 May 8;20(5):e0323103. doi: 10.1371/journal.pone.0323103 (PMC12061115; doi:10.1371/journal.pone.0323103)
Supplement: Attachment — Submitted filename: Response to Reviewers.docx [file pone.0323103.s004.docx]

**PONE-D-25-01605: Response to Reviewers**

Note: line numbers refer to the revised manuscript without track changes.

| **Reviews** | **Response** |
| --- | --- |
| Editor-a: Germany, unlike the United States, suffered the WW1 structural shock on its infrastructure, something the US didn't suffer, but France and the UK also suffered - if the WW1 shock is persistent, then not only Germany, but also France and the UK should experience decline. But I understand that would be an entirely new hypothesis, so it can be a recommendation for future research; however, besides Crawford's study, is there any further studies that use WW1 as a variable? I noticed that WW1 is referenced only once in the manuscript, and WW2 is referenced more; so, given your dataset and analysis, is it possible to say that WW1 did not play a big role in German decline (which started around 1934, according to your manuscript)? | We carefully examined our dataset for a possible effect of WW1 on the proportion of nominators and nominees by country. To do this, we divided data into two periods: one before WW1 (1901-1914) and one after WW1 (1919-1933) but before the political regime change in Germany leading up to WW2.  Our analysis shows that the share of German nominators remained stable at around 34% in both periods, suggesting that WW1 did not have an immediate negative impact on Germany's ability to participate in the nomination process. Among nominees, Germany's share actually increased from 31% to 43%, contradicting the hypothesis that WW1 significantly weakened its scientific standing. Meanwhile, in the US, the share of both nominators and nominees almost doubled between these periods, signalling their growing influence.  Interestingly, a decline possibly linked to WW1 can be observed in the UK, where the share of nominators fell from 7% to 4% and the share of nominees from 15% to 5%. France, on the other hand, showed more stability, with the share of nominators remaining at 15% and the share of nominees falling only slightly (from 24% to 18%). These patterns suggest that WW1 may have had different effects in different countries, but in the case of Germany the evidence does not suggest a strong impact directly on nominations.  A possible explanation for Germany’s sustained role in the nomination process after WW1 is the influence of highly networked scientific elites, who, despite increasing nationalization trends, sought to uphold the ideals of an international scientific community and to maintain its transnational ties (Friedman 2001, pp. 87, 99, 111, 130, 217). For example, Svante Arrhenius, a key member of the Physics Nobel Committee, maintained close ties with the German research elite and actively worked to maintain Germany's leading role in the scientific community.  Furthermore, as Ben-David has noted, the German university system had structural weaknesses well before WWI, but its global reputation and the the continued acknowledgment of German universities as leading institutions within the scientific community helped to uphold its standing. These factors may have mitigated the potential negative impact of WW1 on German nominations.  In terms of previous research on this topic, one study addressed the impact of WWI on the scientific reward landscape/ Nobel Prize nomination data. Iaria et al. (2018) investigates whether WW1 affected groundbreaking research. Using Nobel Prize nomination data 1905-1945 together with citation and patent data, they find that WW1 significantly disrupted knowledge flows, particularly harming highly cited and internationally connected scientists. Their results suggest that Germany and Austria(-Hungary) were disproportionately affected, leading to a measurable decline in groundbreaking research from these countries.  In summary, while our dataset does not show a clear decline in German nominations after WW1, the existing literature suggests a broader disruption in international scientific collaboration based on a broader dataset on scientists and publications. We acknowledge this discrepancy and have incorporated these findings into our paper (lines 436-454, 630-644). |
| Editor-b: I would recommend against including the Economics Nobel for future research (line 532), because it is technically not a "real" Nobel, and it was established in 1969, when the US-American scientific hegemony was well-established, so it raises another set of questions that might be beyond your research scope (see Avner Offer and Gabriel Sordeberg's The Nobel factor). | We understand the concern regarding the Economics NP and agree that its inclusion raises additional questions beyond the scope of our study. We have therefore removed this passage from the paper. |
| Editor-c: As additional references, I can suggest recent work by W. MacLeod and M. Urquiola's "Why Does the United States Have the Best Research Universities? Incentives, Resources, and Virtuous Circles" and J. Dittmar and R. Meisenzahl's "The Research University, Invention and Industry: Evidence from German History" - they are optional, but they are also recent work. | Thank you for these helpful references. We have incorporated them into our manuscript (see lines 188-193, 201-203, 227-229, 327-332, 565-572).  Dittmar and Meisenzahl provide valuable historical context for the deep-rooted link between German universities, knowledge production and industrial innovation. Their study shows that by the mid-1800s, German universities played a central role in fostering scientific and technological progress, with measurable effects on industrial growth.  In the context of our study, this historical trajectory helps to explain why German scientists and institutions have continued to receive significant recognition in the Nobel Prize nomination process, even amidst political disruptions.  We further elaborate on how Urquiola’s perspective align with and support our findings in our response to Rev. 1. |
| Rev. 1: At the same time, they paper exaggerates the novelty of its findings. […]  1) The authors of state that "in contrast to previous methodologies", their " approach utilizes the actual country where the scientist was employed at the time of nomination."  2) More importantly they state that "the transition in leadership between Germany and the United States during the first half of the 20th century represents a significant finding."  In fact, the latter finding was already extensively documented by Urquiola (2020) in a book published by Harvard University Press “Markets, minds, and money”. That book documents this transition and does so using data on where scientists were employed and/or trained rather than nationality. […] the key thing the paper needs to do is to say how it's findings are different from those in the book. | We have revised our explanation of the methodological approach (lines 320-324) to clarify that our reference to “previous methodologies” specifically concerns prior studies on Nobel Prize nominations. We do not intend to overstate the novelty of our approach; rather, we aim to highlight that our dataset offers a particularly precise and comprehensive account of the actual country and institutional affiliation of every individual involved in the Nobel Prize nomination process as nominator and/or nominee in Physics and Chemistry up until 1969. Our dataset includes 3,319 individuals and 8,832 nominations, enabling us to track annual changes in scientific affiliations with accuracy.  Previous studies on Nobel Prize nominations have taken different approaches to defining national affiliation. For example, Gallotti and De Domenico (2019) apply a majority rule for country affiliation but do not specify how it is determined. Crawford (1992) introduced an eight-year threshold for a change in residence but does not justify this specific time frame. In contrast, our approach does not rely on predefined benchmarks but instead records the actual country of employment at the time of each nomination.  We are grateful to the reviewer for drawing our attention to Urquiola's work, which offers an important perspective on the shift in scientific leadership. Indeed, our findings are consistent with his and we appreciate the opportunity to position our study more explicitly in relation to this research. However, it is important to clarify the differences in our approaches. Urquiola (2020) focuses on the career stages of laureates, examining where they were trained and employed. Heinze et al. (2020) take a similarly detailed look at laureates, tracing their affiliations during their PhD, Nobel research, and Nobel award related career steps. Both studies provide valuable insights into long-term institutional prestige and historical patterns. However, while laureate studies capture the culmination of scientific recognition, they do not take into account the processes that shape scientific influence before a prize is awarded.  Our study addresses this gap by shifting the focus from laureates to the nomination process, which is a crucial yet often overlooked stage in the scientific recognition system. The nomination process reflects contemporary assessments of scientific excellence and institutional prestige at a given moment in time, rather than the retrospective recognition embodied in laureate data. By analyzing nominations, we gain a more immediate and dynamic perspective on how scientific influence is distributed globally before it is ultimately consolidated in Nobel awards.  Generally speaking, our data on nominees and nominators confirm the findings of Urquiola (and Heinze et al.). In particular, Tables A.2 and A.3 of Urquiola show that the USA overtook Germany in the number of mentions in Nobel laureate biographies for the first time in the period 1921-1940. This period is also the focus of our analysis, where we examine the structural shifts within nomination data and conclude that the early 1930s is a crucial turning point. This is consistent with Urquiola's findings. The fact that different methodological approaches converge on the same general trend strengthens the robustness of our results. |
| Rev. 2-a: Drawing on the nomination database, the authors discuss centers and peripheries in science. As I understand them, they use the nominations as a “real-time” perspective, but is this really the case? Do not nominators propose candidates for the award that in most cases did their research decades ago? How can the authors take this time-lag into account? | We appreciate the reviewer’s question regarding the potential time lag between scientific achievement and Nobel Prize nominations. To address this concern, we conducted an analysis comparing the time span between a laureate’s first nomination and their eventual receipt of the prize within our dataset. Our findings indicate that, on average, the time span between the first nomination and the receipt of the award is about seven years. This delay increases only slightly over time, with an average of five years in the period 1901–1910 and seven years in the last nomination period (1960–1969).  Our results align with existing research on the growing time gap between scientific discoveries and Nobel Prize recognition (Becattini et al. 2014; Fortunato 2014; Polemis & Stengos 2022). Mitsis (2022) further highlights that the waiting period for the science prizes (Physics, Chemistry & Medicine) has increased over time, with an average of 18 years between pioneering work and prize recognition. Within our observation period up to 1969, this delay is notably shorter: on average, 14 years for Chemistry and 13 years for Physics (Mitsis 2022, Table 2).  Crucially, our analysis reveals that the time span between first nomination and prize receipt (on average, seven years) is significantly shorter than the time gap between the original scientific achievement and the award (13–14 years for Chemistry and Physics). This suggests that while nominations do not capture discoveries in real time, they occur when contributions have already diffused into the scientific community and are actively recognized as prizeworthy.  In comparison to laureate recognition, the nomination process thus serves as a more immediate reflection of contemporary scientific prestige. While laureates are often honored for work conducted decades earlier, nominations capture active debates and recent advancements. Moreover, nominations encompass a broader pool of prizeworthy discoveries, including many outstanding contributions that ultimately do not receive the award. As Zuckerman (1977) notes, the scientific elite is not limited to Nobel laureates but extends to a "41st chair"—the many researchers who meet the highest standards of excellence but are not among the select few awarded the prize. By examining nominations, we gain insight into the broader landscape of recognized excellence, rather than just the final recipients of the prize.  To address this point, we have revised our manuscript and included this discussion in lines 126-135. |
| Rev. 2-b: State of research: During the last couple of years, Seeman and Restrepo have analyzed the nomination database with a focus on chemistry (e.g. https://pubmed.ncbi.nlm.nih.gov/37204108/) I can also recommend the work by Jacob Habinek on nomination networks in science  (Minor note, perhaps there is more recent work than Hollingsworth, Merton and Ben-David) | We appreciate the reviewer’s reference to the work of Seeman & Restrepo and welcome the opportunity to clarify our methodological approach in comparison.  Seeman & Restrepo’s studies provide valuable qualitative insights into the Nobel nomination process by analyzing selected case studies of both successful and frequently overlooked nominees such as Christopher Ingold. Their approach highlights individual nomination trajectories and sheds light on the influence of professional networks and biases in the selection process. Similarly, studies from Nils Hansson’s group explore historical and contextual aspects of nominations for certain individuals (for example, Lady May Mellanby and Walter Hess as precedents for dental research in Hense et al. 2022). As these study focus primarily on qualitative case analyses, they do not aim to provide a systematic, quantitative assessment of global nomination trends.  Our study, in contrast, employs a quantitative approach to analyze the full set of available nomination data. This allows us to draw broader conclusions about geopolitical shifts and structural patterns in scientific recognition over time. While qualitative case studies offer detailed context, they may be influenced by other biases and cannot always be generalized. By systematically analyzing the complete dataset, we aim to provide a comprehensive, data-driven perspective on how nominations reflect and shape the evolving landscape of scientific excellence.  We advocate for a methodological approach that combines large-scale quantitative analysis with historical context to more effectively capture the underlying dynamics of Nobel Prize nominations—an approach also reflected in Habinek’s study on the Literature Nobel Prize (2023) and in Habinek and Fu’s (2023) research on social and cultural closure in Physics Nobel Prize decisions. To clarify this distinction, we have incorporated a discussion of methodological differences in lines 106-123 of our manuscript.  Thanks to the reviewers’ insightful comments, we have integrated additional recent sources that further enrich our theoretical framework. |
| Rev. 2-c: Terminology: I do not see a clear definition of what the authors mean with self-nominations, please clarify. My first assumption would be that it is about scholar X nominating himself, but here the authors, I guess, have another definition. Zuckerman and Crawford talk about “favourite sons” (Poincaré), when a scholar is nominated by scholars who work in the same country, see for example Hansson and Schagen on Sauerbruch in NTM or about Nobel nomination campaigns in Hansson and Schlich in Notes and Records | We appreciate the reviewer’s request for clarification regarding our definition of self-nominations. As correctly pointed out, the term could be misleading if interpreted as an individual nominating themselves, which is explicitly prohibited by the Nobel Prize Statutes. Instead, we refer to a broader concept of self-nomination, where scientists nominate colleagues from their own organizational or national affiliation—essentially the closest possible form of self-nomination within the given rules.  This practice aligns with broader patterns of academic self-reproduction, such as universities hiring their own PhD graduates, which has been shown to be more prevalent among high-status institutions (Wapman et al., 2022). It reflects mechanisms of social closure and elite cohesion, where established networks reinforce their own members' visibility and prestige.  Elisabeth Crawford’s seminal work (1992, 2001) categorized such nominations as self-nominations on the national level (“own-country”), interpreting high levels of self-nominations as chauvinistic and problematic with regard to the universalism norm. However, we find this classification somewhat problematic. The distinction between particularistic and universalistic nominations is not always clear-cut: a scientist nominated by fellow nationals may well be a deserving candidate based on scientific excellence, just as a cross-national nomination could be motivated by informal personal networks rather than objective merit. The example of Henri Poincaré illustrates this tension—he was frequently nominated by French colleagues (seemingly a “favorite son”), yet also widely recognized internationally and nominated across multiple countries (namely from Germany, Italy, Japan, Russia, Sweden, Switzerland, Spain, the Netherlands, UK, and USA).  Given these ambiguities, we approach self-nominations as a sociological phenomenon that reflects underlying structures of scientific hegemony rather than making normative judgments about their legitimacy. Instead of categorizing them strictly as meritocratic or particularistic, we consider them in the context of how dominant scientific actors consolidate influence and maintain epistemic control. Patterns of self-nomination can thus be seen as part of broader stratification mechanisms that shape which scientific communities gain international recognition and which remain at the margins. To clarify this distinction, we have expanded our discussion of self-nominations in lines 264-286 of our manuscript. |
| Have the authors made all data underlying the findings in their manuscript fully available?  Reviewer #2: No | We would like to briefly address this point, although we did not find an explicit comment on it (we hope we have not overlooked anything). Our data and analysis are stored on the OSF platform. As stated during submission, the data was set to be made publicly available upon publication (this requires only a simple setting change in OSF). During the review process, the data has been fully accessible via the view-only link provided with the submission.  To ensure full transparency, we have now made the data publicly available as part of this revision. We hope this resolves any concerns and appreciate the opportunity to clarify. |
